# Chinese Herbal Medicine for Myelosuppression Induced by Chemotherapy or Radiotherapy: A Systematic Review of Randomized Controlled Trials

**DOI:** 10.1155/2015/690976

**Published:** 2015-02-23

**Authors:** Youji Jia, Huihui Du, Min Yao, Xuejun Cui, Qi Shi, Yongjun Wang, Yanping Yang

**Affiliations:** ^1^Longhua Hospital, Shanghai University of Traditional Chinese Medicine, Shanghai 200032, China; ^2^Institute of Spine, Shanghai University of Traditional Chinese Medicine, Shanghai 200032, China

## Abstract

*Background*. Myelosuppression is one of the major side effects of chemo- and radiotherapy in cancer patients and there are no effective interventions to prevent it currently. Chinese herbal medicine (CHM) may be helpful due to its multidrug targets.* Objectives. *This study was designed to evaluate effectiveness of CHM on preventing patients from experiencing myelosuppression by chemo- or radiotherapy.* Search Methods. *Randomized controlled trials (RCTs) were retrieved from seven different databases from the date of database creation to April 2014. We assessed all included studies using Cochrane Handbook for Systematic Reviews of Interventions 5.1.0 and performed statistical analysis using RevMan 5.2.1.* Results*. Eight RCTs were included (818 patients). Pooled data showed that increase of white blood cells (WBCs) is higher with CHM plus chemotherapy/radiotherapy than with chemotherapy/radiotherapy only. Both CHM compared to placebo and CHM combined with chemotherapy/radiotherapy compared to chemotherapy/radiotherapy lacked significant differences in the peripheral platelets, red blood cells (RBCs), and hemoglobin changes.* Conclusions. *Our results demonstrated that CHM significantly protected peripheral blood WBCs from a decrease caused by chemotherapy or radiotherapy. There were no significant protective effects on peripheral RBCs, hemoglobin, or platelets, which may be related to low quality and small sample of included studies.

## 1. Introduction

Myelosuppression, also known as bone marrow suppression or myelotoxicity, is a decline in the activity of the bone marrow, resulting in decreased numbers of WBCs, platelets, and RBCs. Myelosuppression is one of the most commonly observed side effects of chemotherapy and radiotherapy, and it is also a listed side effect of many chemotherapy drugs. Patients are usually given these medications anyway because dying from cancer poses a more immediate threat. Therefore, the possibility of myelosuppression must be considered and monitored when using a chemo- or radiotherapy treatment plan.

Once patients undergo myelosuppression, the bone marrow cannot make the normal level of blood cells. Given that many blood cells have a very short life in the body, patients start to suffer medical complications almost immediately. These include anemia from a low number of RBCs, hemorrhage due to thrombopenia, and immunosuppression caused by a low number of WBCs. Patients will be at risk of developing fatal infections and will not be able to fight them off [1–8], which contributes to the survival rate of the malignancies.

It is crucial to avoid damaging nonmalignant cells during the clinical application of chemotherapy and radiotherapy to reduce morbidity and mortality from infections due to myelosuppression. There have been many research attempts to find safe agents that can reduce myelosuppression and improve the immune response in chemotherapy- or radiotherapy-treated patients. One treatment that has become increasingly attractive in recent years is the use of alternative therapies, especially CHM, as an adjunctive treatment to prevent myelosuppression. Numerous studies have already reported the myelosuppression reduction effects in cancer patients who received CHM during their chemotherapy or radiotherapy treatment. These studies had a variable design and have generally reported inconclusive or conflicting results, making the clinical decision of whether to recommend or omit the use of CHM during chemotherapy/radiotherapy in cancer patients difficult [9–12].

It would be worthwhile to assess the quality and evaluate the efficacy of data from trials according to the principles and measurements of evidence-based medicine. There is no previously published systematic review examining the role of CHM to prevent myelosuppression caused by chemotherapy or radiotherapy. In the present study, we sought to perform a systematic review of RCTs on the use of CHM during chemotherapy or radiotherapy of cancer patients to generate a more precise estimate of the possible therapeutic value of CHM on preventing myelosuppression.

## 2. Materials and Methods

### 2.1. Inclusion Criteria

#### 2.1.1. Study Design

Our review was restricted to RCTs that compared CHM plus chemotherapy/radiotherapy with placebo plus chemotherapy/radiotherapy or chemotherapy/radiotherapy alone.

#### 2.1.2. Participant Characteristics

We included all patients with any type of solid tumor or hematologic malignancy, who accepted chemotherapy or radiation therapy combined with CHM, irrespective of the patient's sex, age, ethnicity, and occupation. All appropriate definitions of myelosuppression included decreased peripheral blood WBCs, RBCs, platelets, or hemoglobin. Patients with serious medical conditions were excluded.

#### 2.1.3. Types of Intervention

The intervention was required to be a clinical trial evaluating all forms of CHM (herbal formula, single herb, herbal extractions, or compounds including herbs and other supplements), which were administered either orally or intravenously, used alone or in combination with other herbs for subjects in the treatment and placebo groups or without additional intervention except chemotherapy or radiotherapy in the control groups.

#### 2.1.4. Outcome Measures

The outcome measures included changes in the peripheral blood WBCs as the primary outcome and changes in the peripheral blood RBCs, platelets, and hemoglobin as the secondary outcomes.

#### 2.1.5. Methodological Quality Assessment

The methodological quality of all included trials was independently assessed by two reviewers according to “Risk of Bias table,” which is recommended by Cochrane Handbook 5.1.0. Reviewers were not blinded with respect to the authors, institution, and journal because they were familiar with the literature. Two review authors (Youji Jia and Huihui Du) independently assessed the risk of bias with the criteria in the Cochrane Handbook for Systematic Reviews of Interventions 5.1.0 (http://www.cochrane-handbook.org). Random sequence generation (selection bias), blinding of participants and personnel (performance bias), allocation concealment (selection bias), blinding of outcome assessment (detection bias), incomplete outcome data (attrition bias), selective reporting (reporting bias), and other sources of bias were scored as “yes,” “no,” or “unclear” according to the definitions of each of the criteria. Disagreements between review authors were resolved by discussion or with a third author (Xuejun Cui). The methodological quality assessment of the trials was used to exclude trials with fatal flaws, such as a dropout rate higher than 50%.

### 2.2. Exclusion Criteria

Exclusions included case or experience reports, preclinical studies (e.g.,* in vitro* and animal studies), review, systematic review, trials in which the treatment groups included ingredients not considered CHM (such as acupuncture, massage, and exterior use), nonrandomized controlled trials, and publications without original data on the outcomes.

### 2.3. Search Methods

#### 2.3.1. Data Sources

We searched PubMed, EMBASE, the Cochrane Library, the CNKI, Wanfang Data, the VIP, and the CBM, a total of 7 electronic databases, from the date of database creation to April 2014.

#### 2.3.2. Searching Key Words

We followed the research strategy using the following key words: (I) Neoplasm OR Tumor OR Neoplasia OR Cancer; (II) Chemotherapy OR Pharmacotherapy OR Adjuvant Drug Therapy OR Drug Polytherapy OR Drug Therapy; (III) Radiosurgery OR Radiotherapy OR radiation therapy OR radioisotope therapy OR X-ray therapy OR Radioisotope Brachytherapy OR Radioisotope Plaque Therapy OR Curietherapy; (IV) Erythrocyte OR Red Blood Corpuscle OR Red Blood Cell OR Erythrocytopenia OR Erythropenia OR Anemia; (V) Granulocytopenia OR Agranulocytosis OR Granulopenia OR White Blood Corpuscle OR Leukocyte OR White Blood Cell; (VI) Soterocyte OR Platelet OR Haematoblast OR Thrombocyte OR Thrombocytopenia OR Thrombopenia; and (VII) (trial OR randomly OR clinical trials OR placebo OR randomized OR randomized controlled trial OR controlled clinical trial) NOT (animals NOT humans), regardless of the language and publication status.

### 2.4. Data Collection and Statistical Analysis

#### 2.4.1. Study Selection

Two independent reviewers (Youji Jia and Huihui Du) evaluated the title and abstract of every document retrieved from the literature searches. The full text of all potentially relevant articles was explored in any language. For confusing or missing information, we contacted the authors, where possible. For duplicate publications, the original one was used.

#### 2.4.2. Data Extraction

Two reviewers (Youji Jia and Huihui Du) independently extracted the study characteristic data from all eligible articles, including the authors, publication date, study type, participants, sample size, interventions, outcomes, baseline treatment, type of CHM, and follow-up. The authors were contacted for more information, as needed. Two review authors (Min Yao and Xuejun Cui) checked and entered data into Review Manager (RevMan 5.2.1).

#### 2.4.3. Statistical Analysis

Statistical analysis was performed using RevMan 5.2.1. The results were pooled and continuous data were expressed as the weighted mean difference (WMD) or standardized weighted mean difference (SMD) with a 95% CI.

The chi-square test (*x* ^2^ test) and *I* ^2^ statistic (*I* ^2^ stands for the percentage of variability owing to between-study variability) were used to evaluate the heterogeneity of intervention effects. The clinically and statistically homogeneous studies were pooled using the fixed effect model if *P* > 0.05 (*I* ^2^ ≤ 50%), when it was considered to have better homogeneity. The clinically homogeneous and statistically heterogeneous studies were pooled using the random effects model if *P* ≤ 0.05 (*I* ^2^ > 50%), when there was heterogeneity between studies. The results of meta-analysis were described graphically using the forest plot. Subgroup analysis was performed based on the clinical heterogeneity, such as type of CHM used.

Funnel plots were made to assess the publication bias, when at least 10 trials were included in the meta-analysis.

## 3. Results

### 3.1. Description of the Included Studies

**Figure 1:**
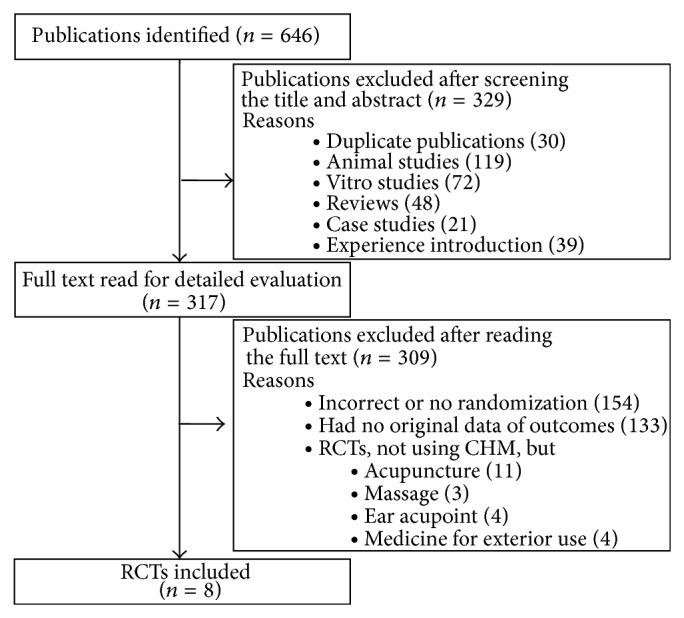
Flow of the included studies.

In total, 646 articles were retrieved following the search strategy described above (259 in English and 387 in Chinese). Potential studies, including 14 in English and 303 in Chinese, were identified by title and abstract screening to exclude trials that were duplicates [13, 14], reviews [15–17], animal studies [18–20], and case or experience reports [21, 22]. By reading the full text we excluded those studies with incorrect randomization or lack of randomization [23–25]; those studies that lacked original data of outcomes [10, 26–28]; and RCTs using acupuncture, massage, ear acupoint, medicine for exterior treatment [29–31]. Eight trials met the inclusion criteria (see the details in Figure 1) and were included in the final review. Two of the trials were published in English, and six of them were published in Chinese. Included studies were published from 2001 to 2013.

These eight trials were all RCTs using CHM, and the duration of studies ranged from 1 to 3 years. Six of the studies were performed in mainland China, and two of them were conducted in Taiwan.

A total of 818 subjects (429 males and 389 females) were included in the eight trials. The number of patients included in each study ranged from 58 to 235, and there was an average sample size of 103.5. There are seven adult patients and one pediatric patient, who had a total of 13 different types of cancer, including breast cancer, colon cancer, nasopharyngeal cancer, lung cancer, colorectal cancer, stomach cancer, leukemia, esophageal cancer, pancreatic cancer, prostate cancer, neuroblastoma, Wilms tumor, and hepatoblastoma. The baselines of all eight randomized studies were compared between the treatment and control groups, and there were no statistically significant differences.

The intervention varied noticeably across the trials. All eight trials included a basic chemotherapy or radiotherapy in both the test and control groups and five of the trials described the type of chemotherapy drugs used. Two [32, 33] of the trials included placebo in the control group and the remaining six trials did not include any other intervention except basic chemotherapy or radiotherapy, allowing for comparison between the CHM (test) and control groups. For the test groups, three of the trials used decoction of the CHM formula [34–36], three of the trials used Chinese patent medicine (particle or soluble granules) [37–39], and two [32, 33] of the trials used extracts of CHM (Table 1).

All eight trials showed routine blood reports, including the WBCs, RBCs, and hemoglobin (Hb) and platelet (PLT) values.

### 3.2. Risk of Bias in the Included Studies

**Figure 2:**
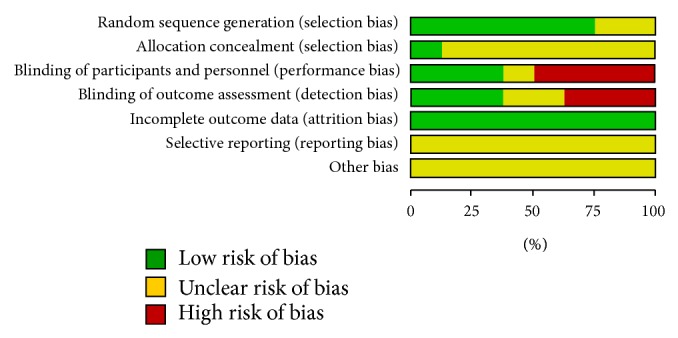
Risk of bias graph: review authors' judgments about each risk of bias item presented as percentages across all included studies.

The reports of all trials mentioned randomization, but only five described the method of randomization [35–39]. In addition, the reports of three trials mentioned double-blinding [32, 33, 38]. We assessed all included studies according to the Cochrane Handbook for Systematic Reviews of Interventions 5.1.0.  Figure 2 shows the results of the author's judgment about each methodological quality item for each included study. One of the studies was defined as medium quality and the others were of low quality.

### 3.3. Effects of the Interventions

#### 3.3.1. Effects of Chinese Herbal Medicine Combined with Chemotherapy/Radiotherapy versus Chemotherapy/Radiotherapy on Protecting the White Blood Cells in Cancer Patients Undergoing Chemotherapy or Radiotherapy

**Figure 3:**
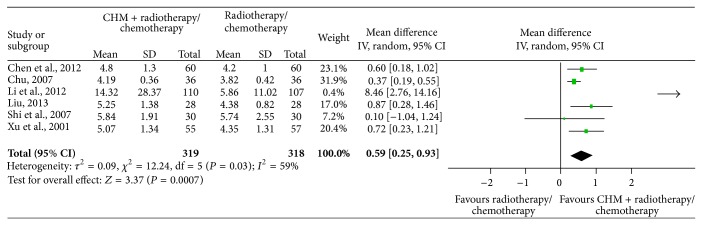
Meta-analysis of studies evaluating the effect of CHM on preventing WBC loss in cancer patients undergoing chemotherapy or radiotherapy.

**Figure 4:**
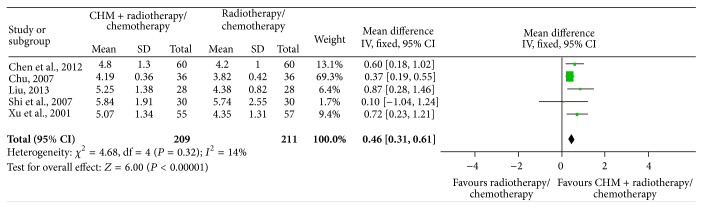
Meta-analysis of studies evaluating the effect of CHM on preventing WBC loss in cancer patients undergoing chemotherapy or radiotherapy after one study was dropped.

There were 6 RCTs [34–39] that studied the protective effects of CHM combined with chemotherapy/radiotherapy versus chemotherapy/radiotherapy treatment alone on decreasing the WBCs in clinical cancer patients. The studies were combined in a meta-analysis and showed a high degree of heterogeneity (Chi^2^ = 12.24, *P* = 0.03, *I* ^2^ = 59%) with a total of 319 subjects in the intervention arm and 318 in the control arm. The high heterogeneity may be caused by the existing issues with WBC examination and the SD value in one of the studies performed by Li et al. 2012 [38], which was obviously different from the others, while the change of increased WBCs is higher in patients treated with CHM than placebo at 0.59 (95% CI: 0.25 to 0.93) (Figure 3). When this study was excluded for the sensitivity analysis, the degree of heterogeneity dropped (Chi^2^ = 4.68, *P* = 0.32, *I* ^2^ = 14%), resulting in 209 patients in the intervention arm and 211 in the control arm. The overall effect estimate continuously showed a significant trend, supporting the treatment of CHM at 0.46 (95% CI: 0.31 to 0.61) (Figure 4).

#### 3.3.2. Effects of Chinese Herbal Medicine on Protecting Red Blood Cells from Decreasing in Response to Chemotherapy or Radiotherapy in Cancer Patients

**Figure 5:**
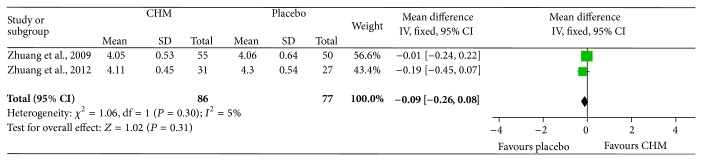
Meta-analysis of studies evaluating the effect of CHM on preventing RBCs from decreasing in cancer patients undergoing chemotherapy or radiotherapy.

Two trials that included an RBC examination conducted a placebo-controlled test and were combined in a meta-analysis (Figure 5), including a total of 86 patients in the intervention arm and 77 in the control arm. There was very little heterogeneity between the studies (Chi^2^ = 1.06, *P* = 0.30, *I* ^2^ = 5%) and there were no significant differences in the RBCs between CHM and placebo when used during chemotherapy or radiotherapy in clinical cancer patients, with a value of −0.09 (95% CI: −0.26 to 0.08).

Only one included study investigated the change in the RBCs between CHM combined with chemotherapy and chemotherapy, which also showed no statistically significant difference (*P* > 0.05).

#### 3.3.3. Effects of Chinese Herbal Medicine on Protecting Platelets from Decreasing in Cancer Patients Undergoing Chemotherapy or Radiotherapy

**Figure 6:**
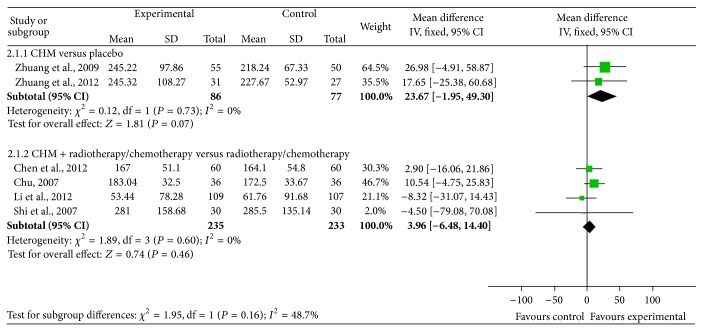
Meta-analysis of studies evaluating the effect of CHM on preventing platelets from decreasing in cancer patients undergoing chemotherapy or radiotherapy.

Six reports with platelet measurements were divided into two subgroups. One subgroup included two studies that compared the effects of CHM versus placebo during chemotherapy or radiotherapy in clinical cancer patients [32, 33], including a total of 86 patients in the intervention arm and 77 in the control arm. There was no heterogeneity between the two studies (Chi^2^ = 0.12, *P* = 0.73, *I* ^2^ = 0%) and our meta-analysis showed no significant differences in the platelets between the CHM and placebo groups when used together with chemotherapy or radiotherapy in clinical cancer patients, with a value of 23.67 (95% CI: −1.95 to 49.30) (Figure 6, upper part). Another subgroup consisted of 4 studies that compared the effects of CHM combined with chemotherapy/radiotherapy versus chemotherapy/radiotherapy alone on hemoglobin protective effects in clinical cancer patients [34, 35, 37, 38], including a total of 235 patients in the intervention arm and 233 in the control arm. There was no heterogeneity among these studies (Chi^2^ = 1.89, *P* = 0.60, *I* ^2^ = 0%) and the meta-analysis revealed no significant differences in the platelets between CHM combined with chemotherapy/radiotherapy and chemotherapy/radiotherapy alone, with a value of 3.96 (95% CI: −6.48 to 14.40) (Figure 6, lower part).

#### 3.3.4. Effects of Chinese Herbal Medicine on Protecting Hemoglobin from Decreasing in Cancer Patients Undergoing Chemotherapy or Radiotherapy

**Figure 7:**
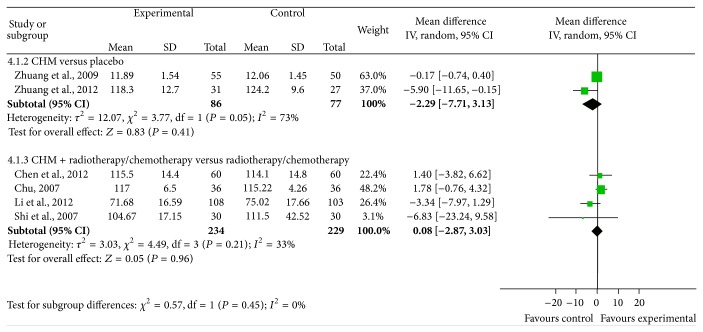
Meta-analysis of studies evaluating the effect of CHM on preventing hemoglobin from decreasing in cancer patients undergoing chemotherapy or radiotherapy.

Six studies included measurements of the serum hemoglobin levels. Two of the studies compared the effects of CHM and placebo during chemotherapy or radiotherapy in clinical cancer patients [32, 33]. These two studies were combined in a meta-analysis and included a total of 86 patients in the intervention arm and 77 in the control arm. There was heterogeneity between the two studies (Chi^2^ = 3.77, *P* = 0.05, *I* ^2^ = 73%) and the effect estimate did not support the CHM intervention, with a value of −2.29 (95% CI: −7.71 to 3.13) (Figure 7, upper part). Four of the remaining studies compared CHM combined with chemotherapy/radiotherapy versus chemotherapy/radiotherapy on the hemoglobin protective effects in clinical cancer patients [34, 35, 37, 38]. These four studies were combined in a meta-analysis and included a total of 234 patients in the intervention arm and 229 in the control arm. There was little heterogeneity among these studies (Chi^2^ = 4.49, *P* = 0.21, *I* ^2^ = 33%) and our meta-analysis revealed no significant differences in the hemoglobin between CHM combined with chemotherapy/radiotherapy and chemotherapy/radiotherapy alone, with a value of 0.08 (95% CI: −2.87 to 3.03) (Figure 7, lower part).

#### 3.3.5. Publication Bias Assessment

Funnel plots could not be performed due to the small number of studies evaluated.

## 4. Discussion

In this systematic review of articles published in English and Chinese, we have identified eight randomized studies using CHM. A total of 818 subjects were included and the duration of studies ranged from 1 to 3 years. Six of these studies were performed in mainland China and 2 of them were conducted in Taiwan. The baselines of these eight randomized studies were compared between the treatment and control groups, and there was no significant difference. Although we searched both English and Chinese databases, we still cannot promise that all relevant trials were found, so the publication bias could not be ignored.

We have tried to identify all RCTs on CHM for prevention of chemotherapy- or radiotherapy-induced myelosuppression, although this might be limited by incomplete citation tracking, as is the case with most systematic reviews. We were able to review studies performed and published in China and English-speaking countries, and a small number of studies performed in Japan and Korea were written in English. We could not include all trials from Korea or Japan written in their native language even though traditional Chinese medicine (TCM) is extensively used in these two countries.

Herbal formulae used in studies performed in China generally showed a good tolerability, while CHM intervention used in studies performed outside China was likely to have more side effects [33]. These differences might be due to the more precise methodology in studies conducted outside mainland China. On the other hand, lack of compliance to the principles of TCM during the selection of herbal formulae may be another reason. In China, the philosophy of TCM emphasizes “personalized therapy,” and the categories of symptoms and signs judged by TCM doctors created the principle for herbal medicine selection. Therefore, even though they have the same clinical diagnosis, different patients may be given different TCM prescriptions depending on the collected symptoms and signs. Additionally, studies performed in China usually do not describe the reasons for falling off, method of randomization, and information on blinding. These methodological limitations may contribute to the better tolerability and lower frequency of adverse effects of CHM in studies performed in China. In spite of these deficiencies, the overall data suggest that CHM was better tolerated [34, 37–39]. Taken together, these preliminary outcomes can form the foundation for designing future trials to assess these therapeutic strategies, preferably by means of rigorous methodologies based on Western principles and selection criteria according to the CHM theory.

CHM generally uses multiple herbs, which may produce complementary and antagonistic effects to balance the benefits and adverse effects. Even with these positive results, some over-the-counter Chinese remedies have been used together with Western medications, which may increase the chance of side effects [40, 41]. These results also underline the importance of quality control and need to standardize the prescribing, dispensing, and administration of these “herbal remedies,” which are often marketed as health supplements without adverse effects.

Given that myelosuppression mainly results from the use of symptomatic therapy, the application of TCM is a possible strategy to address this unmet therapeutic area. Myelosuppression is graded according to anticancer drugs in acute and subacute toxicity of classificatory criteria (WHO criteria) as described in Table 2 [42].

The following is a description of the conventional therapeutic methods. Recombinant human erythropoietin (rhEPO), with supplement of other iron agents (such as dextran), is used to promote erythropoiesis and eliminate the iron utilization obstacle for the RBC thrombocytopenia and anemia. Transfuse RBCs or whole blood when hemoglobin is less than 85 g/L. Reduce movement, control the blood pressure, avoid using antiplatelet drugs, and use interleukin-11 (IL-11) and rhTPO when patients have thrombocytopenia and bleeding. Transfuse blood components with platelets or whole blood when the platelet concentration is less than 20 × 10^9^/L or bleeding is severe. Prevention is preferred for leukopenia/neutropenia, fever, or infection, and we can use conventional drugs to increase the WBCs or hematopoietic stem cell differentiation, promoting the effects of therapy. Apply recombinant human granulocyte colony-stimulating factor (rhG-CSF) to patients with severe symptoms, and use antibiotics to control infection, when necessary. Generally, patients with degree III or higher myelosuppression must be treated. However, there are currently no clear criteria for those belonging to degree II or lower, and treatment mainly focuses on symptomatic therapy [43–45]. However, there is a lack of effective intervention strategies for preventing myelosuppression in the clinic.

This systematic review is based on a number of clinical RCTs, and the quality of included studies was strictly screened and controlled. In this meta-analysis, we concluded that CHM could effectively prevent radiotherapy- and chemotherapy-induced myelosuppression in cancer patients. The reduction in WBC counts during radiotherapy or chemotherapy in cancer patients was blocked by the administration of CHM, which controlled infection. Therefore, the use of CHM is recommended as a basic therapeutic remedy during radiotherapy and chemotherapy in cancer patients to prevent infection due to insufficient WBCs.

Among the six included RCTs that studied the protective effects of CHM on chemotherapy or radiotherapy affected WBCs, it looks like CHM had extremely strong protective effects on WBCs in Li et al.'s report [38], while no protective effects were reflected in Shi et al.'s paper [34], as shown in Figure 3. We found that the type of cancer, age of participants, chemotherapy protocols, and CHM interventions were different between these two RCTs by comparison of the information described in the papers. Most important is that Li et al. studied adults with acute leukemia, while Shi et al.'s report includes children with neuroblastoma, nephroblastoma, and hepatoblastoma; myelotoxicity chemotherapy was used in both RCTs but the forms of CHM used were different. On the first glance it seems that CHM is more effective in treatment of chemotherapy damaged WBCs in adult patients with nonsolid tumors than in children with solid tumors. As we know, acute leukemia is a cancer of primitive WBCs in the bone marrow, characterized by the rapid and overproduction of abnormal WBCs that accumulate in the bone marrow and interfere with the production of normal blood cells. The baseline of peripheral blood WBCs of acute leukemia patients is different from the normal level and different from patients with solid tumors as well. Thus, it is incomparable between the leukemia data and solid tumor data about the protective effects of CHM on chemotherapy or radiotherapy affected WBCs. Also, we could not eliminate the possibility of their miscalculation to include leukemia cells while counting WBCs since this data showed a greater heterogeneity (Figure 3). Therefore, this study was excluded later for a sensitivity analysis in our current meta-analysis, which evaluated the effects of CHM on preventing WBCs loss in cancer patients undergoing chemotherapy or radiotherapy. By reading the paper carefully, we found that the authors stated that the WBCs counts of both groups declined after intervention with CHM, while the CHM group was higher, and the absolute value of WBCs decrease was smaller in the CHM treatment group than in the control group; thus, we came to the conclusion that CHM could protect chemotherapy damaged WBCs. At the same time, similar outcome data in treatment and control group were provided by Shi et al. in this paper, so there are no treatment effects in our analysis. But the baseline data was different between the treatment and control groups provided by Shi et al., which in turn resulted in a treatment effect of CHM. This result is also not credible.

After comparing the detailed information of the four convincing RCTs (Chen and Shen [37], Xu et al. [39], Chu [35], and Liu [36]), we found that common solid tumors of adult patients, such as colon cancer, gastric cancer, lung cancer, and breast cancer, were included in all these studies.* Astragalus membranaceus* and* Angelica sinensis* were used in all their CHM prescriptions, which is a classic coupled CHM for replenishing Qi and Blood. Moreover, tonifying kidney CHM was used in three of the studies, such as sealwort, glossy privet fruit, Radix Polygoni Multiflori, Radix Rehmanniae Preparata, and Fructus Psoraleae. This suggested that tonifying kidney CHM may contribute to treatment of leucopenia caused by myelosuppression.

The higher the quality of the included studies is, the more we can draw scientific conclusions by meta-analysis. Some studies in the literature have limitations. For example, in some, the random method was not clear and blinding was not implemented. Some studies were performed in China or Taiwan, which did not have international registries, and there was a lack of scientific quality control. The same group in Taiwan performed two of the studies, which may have a performance bias. The included studies had heterogeneity in the type of cancer and use of CHM; as a result, the subgroup analyses could not be conducted. These may, to some extent, limit the scientific validity of the analyzed results.

## 5. Conclusions

In conclusion, we demonstrated that CHM significantly prevented peripheral WBCs from being damaged by chemotherapy and radiotherapy in cancer patients by comparing CHM plus chemotherapy or radiotherapy with chemotherapy or radiotherapy alone. However, these results provide no convincing evidence for the efficacy of CHM on recovering platelets, red blood cells, and hemoglobin, which were affected by chemotherapy and radiotherapy in cancer patients. However, this may be due to the small number, size, and methodological quality of the available RCTs that used CHM to prevent bone marrow suppression as a result of radiotherapy and chemotherapy. Further rigorous, multicenter RCTs with a large sample size are necessary to further examine these topics, but they must overcome the limitations present in the current publication. This will benefit patients with decreased bone marrow function.

## Figures and Tables

**Table 1:** Details of the included studies.

Author, year	Study types	Participants	Sample size (T/C)	Intervention	Outcome	Baseline treatment	Detail content of CHM	Lost to follow-up
Zhuang et al., 2009 [32]	Randomized, double-blind	Breast/nasopharyngeal/colon/lung	55/50	Extract of CHM versus placebo	PLT, RBC, and Hb	Chemotherapy or radiotherapy	*G. lucidum* extract, *C. pilosula* extract, *A. sinensis* extract, and citronellol powder were mixed into a capsule	16 incomplete, 7 giving up, 1 and poor compliance

Zhuang et al., 2012 [33]	Randomized, double-blind	Breast cancer	31/27	Extract of CHM versus placebo	PLT, RBC, and Hb	Chemotherapy or radiotherapy	*A.sinensis* extract, *C. pilosula* extract, *G. tsugae* extract, rose geranium power were mixed into a capsule. Liquid extracts were obtained following extraction with boiling water for 1 h. The extracts were freeze-dried	6 therapeutic regimen changed, 1 erythra, and 1 intolerance in capsules taste

Chen and Shen, 2012 [37]	Randomized	Colon/rectal cancer	60/60	CHM plus chemotherapy versus chemotherapy	WBC, PLT, RBC, and Hb	Chemotherapy	Qisheng Mixture (Astragalus, angelica, Cimicifuga rhizome, and Rhizoma Polygoni Cuspidati) decocted with water into 300 mL	Not described

Xu et al., 2001 [39]	Randomized	Lung/breast/gastric/intestinal cancer	55/57	CHM plus chemotherapy versus chemotherapy	WBC	Chemotherapy	Shuanghuang Shengbai Granule (Astragalus, polygonatum, Glossy privet fruit, Radix trichosanthis, Rhizoma Drynariae, etc.) processed into granules, 15 g per pack	Not described

Li et al., 2012 [38]	Randomized, double-blind	Refractory acute leukemia	118/117	CHM plus chemotherapy versus chemotherapy	WBC, PLT, and Hb	Chemotherapy	Compound Zhebei granules. The preparation method was unknown	Not described

Shi et al., 2007 [34]	Randomized	Neuroblastoma tumors, nephroblastoma, and hepatoblastoma	30/30	CHM plus chemotherapy versus chemotherapy	WBC, PLT, and Hb	Chemotherapy	(1) Millet sprout, rice sprout, Qu Jian, Alpinia katsumadai, Costas, Amomum villosum. (2) Fresh reed rhizome, fresh couchgrass root, Rehmanniae, Cortex Moutan, Agrimony, Platycladus orientalis carbon, and Clematis root decocted with water twice	Not described

Chu, 2007 [35]	Randomized	Lung/breast/gastric/colorectal/esophageal/pancreatic	36/36	CHM plus chemotherapy versus chemotherapy	WBC, PLT, and Hb	Chemotherapy	Bushen Shengxue recipe (Radix rehmanniae, Astragalus root, Angelica, and Radix Polygoni Multiflori) decocted with water	Not described

Liu, 2013 [36]	Randomized	Lung/breast/stomach/prostate	28/28	CHM plus chemotherapy versus chemotherapy	WBC	Chemotherapy	Licorice root, deerhorn glue, Radix Rehmanniae Preparata, Fructus Psoraleae, Himalayan teasel root, Poria, Atractylodes, Radix Pseudostellariae, Angelica, and Astragalus sunburn decocted with water three times into 300 ml	Not described

**Table 2:** Anticancer drugs in acute and subacute toxicity of classificatory criteria (WHO criteria).

Items	0 degree	I degree	II degree	III degree	IV degree
Hemoglobin (g/100 ml)	>11.0	10.9–9.5	9.4–8.0	7.9–6.5	<6.5
WBC (1000/m^3^)	>4.0	3.9–3	2.9–2.0	1.9–1.0	<1.0
Granulocyte (1000/m^3^)	>2.0	1.9–1.5	1.4–1.0	0.9–0.5	<0.5
Platelets (1000/m^3^)	>100	99–75	74–50	49–25	<25
